# Skin Microbiota in Contact Sports Athletes and Selection of Antiseptics for Professional Hygiene

**DOI:** 10.1155/2019/9843781

**Published:** 2019-01-10

**Authors:** Dilyara S. Martykanova, Nailja Ch. Davletova, Ilya A. Zemlenuhin, Venera I. Volchkova, Salavat M. Mugallimov, Azat M. Ahatov, Alexander V. Laikov, Maria I. Markelova, Eugenia A. Boulygina, Leonid V. Lopukhov, Tatiana V. Grigoryeva

**Affiliations:** ^1^Volga Region State Academy of Physical Culture, Sports and Tourism, Kazan, Russia; ^2^Kazan Federal University, Kazan, Russia

## Abstract

**Background:**

The aim of this study was to assess changes in skin microbiota of wrestlers during training sessions and to determine the sensitivity of hemolytic bacterial isolates to antiseptics.

**Methods:**

The main skin bacterial isolates obtained from the skin of 15 wrestlers were identified by cultivation method, with the following MALDI Biotyper and 16S rRNA gene sequencing methods. The sensitivity of hemolytic isolates to antiseptics (Veltosept-2, Cutasept F, Chlorhexidine, Miramistin, and Hydrogen Peroxide) was evaluated by measuring the size of bacterial growth inhibition zone on agar plates.

**Results:**

Opportunistic bacteria of the species* Bacillus cereus*,* Staphylococcus aureus*,* S. epidermidis,* and* S. saprophyticus* were the most commonly found species in skin microbiota of wrestlers before and after training sessions. Representatives of all these species mostly had a hemolytic activity. An alcohol-containing antiseptic Veltosept-2 showed the strongest inhibitory effect on the bacterial isolates of athletes' skin microbiota most frequently detected in this study.

**Conclusions:**

The general increase in the bacterial colonization of wrestlers' skin, as well as the presence of hemolytic forms of opportunistic bacteria in cutaneous microbiota, indicates dysbiotic changes and a decrease in the protective features of the host organism. Veltosept-2 application can reduce the incidence of skin infections in contact sports athletes with the highest efficiency.

## 1. Introduction

At present, considerable attention is paid to the health of athletes and the influence of professional factors on it all over the world. However, almost every athlete during a sports career faces skin problems [1], in particular, the problem of infectious skin diseases [2]. On the one hand, the skin microbiota plays a critical role in the protection of the body, but on the other hand, it is an inexhaustible reservoir of pathogens of exogenous and endogenous infections [3, 4]. In the general structure of infectious pathology of athletes, purulent-inflammatory skin diseases occupy the first place [5]. The infectious skin disease cases resulted from the microbial transmission during training sessions or competitions inevitably lead to the development of pathological conditions that may affect the steady progress in athletes performance [6]. The most common infections found in wrestlers include fungal infections (e.g., ringworm); viral infections (e.g., herpes in wrestlers (Herpes gladiatorum), the causative agent of which is the herpes simplex virus (HSV-1)); bacterial infections (e.g., impetigo) caused by staphylococci, or streptococci, including methicillin-resistant* Staphylococcus aureus* (MRSA) [7–9].

At the moment, the number of works describing this problem significantly exceeds the number of studies devoted to the searching for the most effective antiseptic for the prevention of skin diseases in athletes. Diversity of agents for local treatment complicates the choice due to differences in the mechanism of antiseptic action: some agents work as detergents (Miramistin, Cutasept F), others kill cells by oxidative stress (Hydrogen Peroxide), and third ones denature proteins and block functional groups in the cell wall (Veltosept-2; Chlorhexidine). There is an evidence of the effectiveness of the soap and water wipes used by wrestlers compared to the control group that did not use any means to prevent infectious diseases [10]. There are also some practical instructions for sanitary processing of sports mats [11]. A properly selected regime of hygiene plays an important role in the career of athletes due to the threat of long breaks in competitive and training activities that can lead to the loss of athletic performance. The purpose of this study was to assess the changes in wrestler skin microbiota before and after training sessions and to determine the sensitivity of isolated bacteria to different antiseptics, which can serve as a basis for the selection of the most effective hygienic means to prevent skin diseases in contact sports athletes.

## 2. Materials and Methods

### 2.1. Design of Experiment

Experiment involved 15 wrestlers. The detailed description is given in Table 2. All participants of the research were examined for the presence of various skin rashes, and answered the questionnaire, which included questions about presence or absence of chronic dermatological diseases in their medical history, sports performance, and the number of training hours per week. The study was conducted in December 2016.

### 2.2. Experimental Protocol

The samples were collected before and immediately after two-hour training sessions. The swab method was used in this study. A cotton swab with a diameter of 0.5 cm was moistened in 0.5 ml of physiological saline solution, squeezed on the tube wall, and the skin area of the medial part of the forearms (about 10 square cm) was intensively rubbed by this swab, since this area is the most affected region in wrestlers. The swab was placed in the tube with 1 ml sterile Amies transport medium without charcoal, transported within 24 hours into the bacteriological laboratory, where it was screened on the selective media: 5% blood agar, Egg-Yolk Salt Agar, and chromogenic agar for differentiation of Candida M 1297 A fungi (HiMedia, India). In the same way, the samples were collected from the training mat in several places with a total area of 100 cm^2^ before and after training sessions and cultured on the same media. In 48 hours, the number of microorganisms grown on the sectors was determined. The isolated colonies of microorganisms were identified by the express method using the MALDI Microflex Biotyper device (Bruker, Germany); in difficult cases, Sanger sequencing on a 3730 DNA analyzer (Applied Biosystems, Japan) was used for the identification.

The sensitivity of isolated* Staphylococcus aureus* to oxacillin was assessed using the disc-diffusion method.

The composition of the antiseptic agents used in the research is given in Table 1. Sensitivity of all isolated bacteria to these antiseptics was determined as follows: the test antiseptic in a volume of 10 *μ*l was applied to a culture dish by a sterile tip and after 24 hours of incubation at the temperature of 37°C the diameter of the inhibition zone of bacteria growth in millimeters was measured.

### 2.3. Statistical Analysis

Student's t-test and t-tests with Bonferroni adjustment, respectively, were used to assess the statistically significant differences in the representation of the revealed bacteria and the efficacy of the antiseptics studied.

### 2.4. Ethics Statement

Written informed consent was obtained from all participants prior to the testing. The study complied was approved by the Local Ethical Committee of the Kazan Federal University on June 2 2016 (Protocol N4a).

## 3. Results

The experiment involved 15 wrestlers aged 17-21 years (Table 2). All wrestlers were engaged in national wrestling and belt wrestling: from the first adult level to the master of sports category. The weight of wrestlers was 74.3 kg (± 4.5 kg), height 173.4 cm (± 4.1 cm). The average sport experience of wrestlers was 9.4 (± 3.6) years; the average duration of training per week was 12.6 (± 4.6) h. Eight out of 15 wrestlers suffered from an infectious skin disease at least once every six months. Two athletes had suffered from a chronic dermatitis in the past.

### 3.1. Microbiota of the Training Mat

Two species of microorganisms were found on the mat surface before the training sessions:* Acinetobacter schindleri* (1*∗*10^5^ CFU/cm^2^) and* Pseudomonas stutzeri* (1*∗*10^2^ CFU/cm^2^), none of which were found in skin swabs after training sessions. Among the microorganisms of mats which were detected after training sessions,* Acinetobacter lwoffii* (1*∗*10^5^ CFU/cm^2^),* Staphylococcus aureus* (1*∗*10^5^ CFU/cm^2^),* Staphylococcus epidermidis* (5*∗*10^2^ CFU/cm^2^), and* Micrococcus luteus* (5*∗*10^2^ CFU/cm^2^) were cultivated similar to microorganisms from the wrestlers' skin.

### 3.2. Microbiota of the Wrestlers' Skin

**Figure 1:**
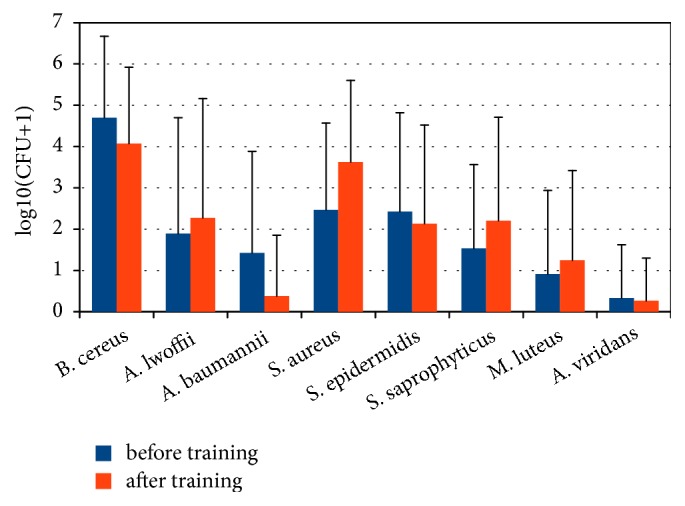
Distribution of the most common representatives of wrestlers' skin microbiota.

Our research revealed that the average occupancy of the wrestler's skin by bacteria is 10^4^ CFU/cm^2^ while the norm for dry skin areas is 10^2^ CFU/cm^2^ [12]. Among the main representatives of microbiota inhabiting the skin, the following microorganisms were detected:* Bacillus cereus, Acinetobacter lwoffii, Acinetobacter baumannii, Staphylococcus aureus, Staphylococcus epidermidis, Staphylococcus saprophyticus, Micrococcus luteus, and Aerococcus viridans *(Figure 1). Their abundance, as well as the significance of differences in their number before and after training sessions, is given in Table 3. On the skin swabs of wrestlers before and after training, fungi of the genus* Candida*, as well as fungi of other genera, were not found.

 Significant differences in the quantitative content of bacteria inhabiting the skin of athletes before and after training were not detected (p> 0.05).

The earliest and most reliable indicator of skin dysbiosis is the presence of hemolytic properties in its microbiota. For example, *β*-hemolytic streptococci are the most common cause of impetigo, erysipelas, and bacteremia [13]. In our research, hemolytic properties were found in the representatives of* Bacillus cereus* species and in all staphylococci (*S. aureus, S. epidermidis,* and* S. saprophyticus*) both before and after training of the athletes. Significant changes in the number of bacterial hemolytic forms after training have also not been detected. The similar situation was observed in our previous study, where the predominance of bacilli in the skin microbiota of wrestlers was confirmed during the metagenomic analysis and their hemolytic properties by cultivation on the selective media [14].

### 3.3. Characterization of the Isolated S. aureus

The sensitivity of isolated* S. aureus* to oxacillin was analyzed. Аll the examples isolated before and after training* S. aureus* were not MRSA.

### 3.4. Analysis of the Sensitivity of Microorganisms to Antiseptics

**Figure 2:**
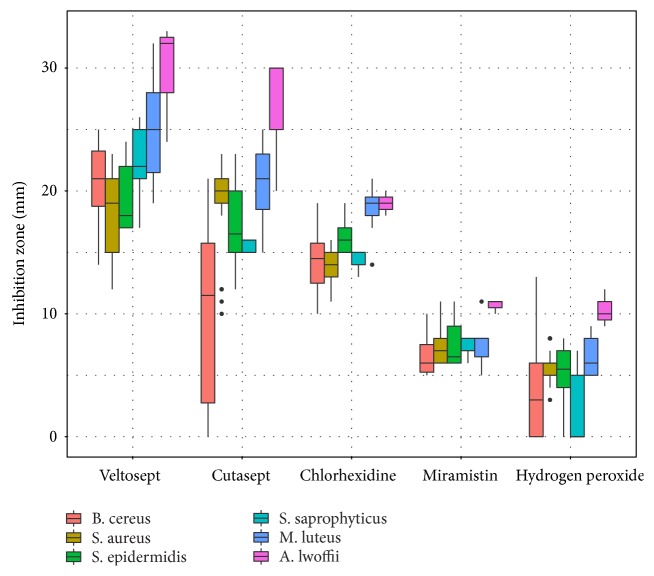
The size of the zones of microorganisms growth inhibition under the influence of the antiseptics studied.

Antimicrobial agents were selected for this research taking into account the convenience of their use in the training and competitive processes (absence of traces on skin after application, rapid evaporation from the surface, hypoallergenicity, and permission to use in medical practice), and also taking into account the difference in their composition and active substance. The effectiveness of the antiseptic was evaluated by the diameter of the bacterial growth inhibition zone on an agar plate. As it can be seen from the Figure 2, antiseptics Veltosept-2, Cutasept F, and Chlorhexidine have the most effective inhibitory effect on microorganisms.

To determine the optimal antiseptic drug which effectively affects the most frequently detected species of bacteria, the significance of differences in the size of the bacterial growth inhibition zone was assessed (Table 4).

Based on significant differences, the drugs studied can be divided into two groups: ineffective and highly effective. Miramistin and Hydrogen Peroxide were considered to be ineffective. Veltosept-2, Cutasept F, and Chlorhexidine were included in the highly effective group. In 48 hours after the plates incubation, secondary growth of* B. cereus* in the inhibition zone with Cutasept F was detected in 33.3% of cases. Cutasept F can not be recommended as a preventive agent. Among the remaining two agents, Veltosept-2 was the most effective. Thus, Veltosept-2 can be recommended for the prevention and treatment of infectious dermatological diseases affecting wrestlers, possible cause of which may be all bacterial species isolated from their skin in this study.

## 4. Discussion

In this research the microbiota of the training mat and the wrestlers' skin before and after training sessions and the sensitivity of the isolated microorganisms to antiseptics were examined.

The obtained data on the bacterial contamination of the training mat indicate that the microbiota of carpets and mats participates in the microbial composition changes in wrestlers before and after training sessions to a lesser extent, and the skin biota of the wrestlers is changed due to the transmission of microorganisms between wrestlers.

The increased amount of skin bacteria may indirectly indicate a decrease of immunity of the examined wrestlers. The weakening of normal physiological processes that determine the inhibitory effect of the body's immunoreactivity on the vital functions of its microbial cohabitants quickly leads to their activation, which manifests itself in an increase in their number even before the development of any pathological processes [15, 16]

Compared with other works [17–19], which described the presence of microorganisms in healthy people's skin, in our research there are differences in abundance of some bacteria in wrestlers. High frequency of occurrence of hemolytic* Bacillus cereus* indicates a general altered skin habitat in wrestlers which more prone to microorganism exposure. Probably as a result of these changes such bacteria as* Acinetobacter lwoffii, Acinetobacter baumannii, and Micrococcus luteus* were identified in our study that normally should not occur.

Recently, an increase in the frequency of hospital infections caused by bacteria of the* Acinetobacter *genus in patients with impaired immunity that is difficult to treat has been reported. These microorganisms are often found in skin lesions and abscesses. The pathogenesis of lesions is directly related to the impaired immunity, since* Acinetobacter* lacks any pathogenic factors, excluding the cell wall lipopolysaccharides [20]. According to [21]* Bacillus cereus* is a widespread saprophyte which can cause human diseases as a part of the microbial associates, especially in individuals with secondary immunodeficiency and immune disorders. It is often impossible to draw a clear boundary between saprophytes and pathogens that are a part of a normal microbiota. In recent years, infections caused by strains of* S. epidermidis*, previously considered to be nonpathogenic, are recorded quite often [22]. Unlimited colonization of the body by any kind of bacteria may give rise to the skin infectious diseases. The leading role in the development of such lesions is played not by the virulence of the pathogen itself, but by the state of the human protective features.

According to the literature, during almost every fight the wrestler gets mechanical damage of the skin (abrasions and cuts), which seriously increases the frequency of the skin infectious disease occurrence [2, 5]. The risk of transmission of cutaneous infectious diseases in wrestling is considered to be the highest in comparison with other sports [10]. This is facilitated by the presence of direct factors (constant “skin to skin” contact of the athletes during the sport activities, noncompliance with the requirements for body hygiene and cleanliness of the uniform and shoes, the presence of athletes with obvious signs of infection at competitions and training) [23], and mediated factors (constant stress as a result of frequent competitions, everyday training, etc.) [24].

To minimize the spread of skin infections and promote the athlete safety, sensitivity of isolated skin bacteria to the most popular antiseptics was tested in our work. The alcohol-containing Veltosept-2 was more effective against most of hemolytic isolates among all antiseptics we studied. In the work of Anderson and coauthors it was shown that the use of different types of sanitary wipes during competitions sharply reduces the risk of developing skin diseases two weeks after contact. The odds of infection compared to the control were 0.089 after soap-water wipes application and 0.44 for 70% of the isopropanol treatment, respectively [10]. It seems that a more gentle soap-based treatment showed better protection the skin of athletes than the more aggressive 70% isopropanol, so the effectiveness of the antiseptics we tested should be checked directly on the skin and tracked the long-term effects on the microbiota as a whole.

The effectiveness of the studied antiseptics will be evaluated on a larger sample and with long-term use, which will allow us to estimate the real contribution of antiseptics to reduce the incidence of skin diseases in athletes.

## 5. Conclusions

The presence of hemolytic forms of bacteria, increased colonization of the skin by bacteria, in particular bacteria of the* Acinetobacter* genus and* Bacillus cereus *species, indicates a dysbiosis of the wrestlers' skin and a decrease in the protective features of the organism in the examined athletes, which are the risk factors for skin infections. Microbiota of training mats was not involved in the change in the wrestlers' skin microbial composition during training sessions.

To prevent and treat the infectious diseases of wrestlers' skin, the best choice are to use the alcohol-containing antiseptics (Veltosept-2; Cutasept F), as well as 0.05% aqueous solution of chlorhexidine bigluconate. Among these antiseptics the Veltosept-2 showed a wide range of antimicrobial activity against most etiological agents isolated from the wrestlers' skin (*B. cereus, S. aureus, A. lwoffii, S. saprophyticus, S. epidermidis, *and* M. luteus*).

## Figures and Tables

**Table 1:** Characteristics of the studied antiseptics.

Name of antiseptic	Composition	Action target
Chlorhexidine	0.05% chlorhexidine bigluconate, purified water	*Trichomonas vaginalis, Neisseria gonorrhoeae, Chlamydia spp., Bacteroides fragilis, Treponema pallidum, Gardnerella vaginalis. Ureaplasma spp.*, moderately active against some strains *Proteus spp.* и *Pseudomonas spp.* Viruses (except the herpes virus) as well as spores of fungi are resistant to the action of the drug.

Veltosept-2	0.1% (±0.01%) clathrate of urea and an quaternary ammonium, 70% (± 3.0%) isopropanol	Gram-positive and Gram-negative bacteria (including tuberculosis pathogens, nosocomial infections), dermatophytes, yeast-like fungi of the genus *Candida*, viruses (including influenza pathogens, including avian influenza, etc. Acute respiratory viral infections, parenteral viral hepatitis, HIV, herpes, rotavirus gastroenteritis, enterovirus infection, hepatitis A, poliomyelitis). Especially dangerous infections (plague, cholera, anthrax).

Cutasept F	0.025% alkyl dimethyl benzyl ammonium chloride, 63% isopropanol	Gram-positive and Gram-negative bacteria, *Mycobacterium tuberculosis*, nosocomial infections pathogens, viruses (HIV, hepatitis B), pathogenic fungi.

Miramistin	0.01% benzyldimethyl-myristoylamino-propylammonium chloride monohydrate, purified water	Gram-positive bacteria (*Staphylococcus spp., Streptococcus spp., Streptococcus pneumoniae*, etc.), Gram-negative bacteria (*Pseudomonas aeruginosa, Escherichia coli, Klebsiella spp.*, etc.), *Aspergillus* and *Penicillium*, yeast (*Rhodotorula rubra*, *Torulopsis glabrata,* etc.) and yeast-like fungi (*Candida albicans*, *Candida tropicalis, Candida krusei, Pityrosporum orbiculare* (*Malassezia furfur*), etc.), dermatophytes (*Trichophyton rubrum, Trichophyton mentagrophytes, Trichophyton verrucosum, Trichophyton schoenleini*, *Trichophyton violacent, Epidermophyton Kaufman- Wolf, Epidermophyton floccosum, Microsporum gypseum, Microsporum canis,* etc.), viruses (herpes viruses, human immunodeficiency virus, etc.), causative agents of sexually transmitted diseases (*Chlamydia spp., Treponema spp., Trichomonas vaginalis, Neisseria gonorrhoeae*, etc.).

Hydrogen peroxide	3% hydrogen peroxide, 0.05% sodium benzoate, purified water	Gram-positive and Gram-negative bacteria.

**Table 2:** Wrestlers description.

ID	Sex	Age, years	Weight, kg	Height, cm	Qualification	Training duration per week, hours	Sports experience, years
1	male	21	80,5	177,5	Candidate for Master of Sport	10	13

2	male	21	73,5	175	Master of Sport	18	11

3	male	21	73	171	Candidate for Master of Sport	6	5

4	male	20	72,9	167,8	Candidate for Master of Sport	14	6

5	male	19	74,1	182,3	Category I	10	12

6	male	17	65,6	169,1	Category I	20	7

7	male	21	75,9	177,6	Candidate for Master of Sport	8	10

8	male	20	69,4	173,2	Candidate for Master of Sport	12	14

9	male	21	84,2	167,5	Master of Sport	12	11

10	male	19	72,5	169,8	Candidate for Master of Sport	14	10

11	male	17	71,7	177,7	Category I	12	1

12	male	21	77,2	171,9	Category I	18	14

13	male	19	71,2	172,9	Candidate for Master of Sport	20	8

14	male	21	77,7	174,6	Candidate for Master of Sport	10	9

15	male	21	75,7	173,7	Candidate for Master of Sport	8	10

**Table 3:** The quantitative content of bacteria found on the athletes skin before and after training sessions.

Microorganisms	Before training			After training			Significance of differences (p)
	Frequency of occurrence, % of cases	Mean, CFU/cm^2^	SD, CFU/cm^2^	Frequency of occurrence, % of cases	Mean, CFU/cm^2^	SD, CFU/cm^2^	
*Bacillus cereus*	86.7	5.8*∗*10^5^	1.25*∗*10^6^	86.7	9.4*∗*10^4^	1.2*∗*10^5^	0.15

*Acinetobacter lwoffii*	33.3	7.47*∗*10^5^	2.57*∗*10^6^	40	2.4*∗*10^5^	3.68*∗*10^5^	0.46

*Acinetobacter baumannii*	26.7	8*∗*10^4^	1.74*∗*10^5^	6.7	3.33*∗*10^4^	1.29*∗*10^5^	0.41

*Staphylococcus aureus*	66.7	2.81*∗*10^4^	4.5*∗*10^4^	80	9.13*∗*10^4^	1.7*∗*10^5^	0.18

*Staphylococcus epidermidis*	53.3	5.27*∗*10^4^	1.29*∗*10^5^	46.7	2.87*∗*10^4^	4.47*∗*10^4^	0.5

*Staphylococcus saprophyticus*	40	9.34*∗*10^3^	2.55*∗*10^4^	46.7	8.17*∗*10^4^	1.73*∗*10^5^	0.12

*Micrococcus luteus*	20	3.34*∗*10^5^	1.29*∗*10^6^	26.7	4.13*∗*10^4^	1.29*∗*10^5^	0.39

*Aerococcus viridans*	6.7	6.67*∗*10^3^	2.58*∗*10^4^	6.7	6.67*∗*10^2^	2.58*∗*10^3^	0.38

**Table 4:** The significance of the differences in the microorganism growth inhibition zones under the influence of the antiseptics studied (p <0.05 is considered as a statistically significant difference with the Bonferroni adjustment).

	Chlorhexidine	Cutasept F	Hydrogen peroxide	Miramistin
Cutasept F	1	-	-	-

Hydrogen peroxide	0.00032*∗*	0.000023*∗*	-	-

Miramistin	0.00503*∗*	0.00035*∗*	1	-

Veltosept-2	0.0412*∗*	0.43001	0.00000014*∗*	0.0000017*∗*

## Data Availability

Authors declare that raw data for this project are stored on the Kazan Federal University servers and available from the corresponding author on reasonable request.
